# A dataset of chronic nicotine-induced genes in breast cancer cells

**DOI:** 10.1016/j.dib.2025.111573

**Published:** 2025-04-22

**Authors:** Samson Mugisha, Shreyas Labhsetwar, Devam Dave, Richard Klemke, Jay S. Desgrosellier

**Affiliations:** Department of Pathology, University of California, San Diego, 9500 Gilman Drive, La Jolla, CA 92093, USA

**Keywords:** Tobacco products, Smoking, Nicotine, Breast cancer, Single-cell RNA-seq, Gene expression, Inflammation

## Abstract

These data show the differentially expressed genes (DEG) from HCC38 breast cancer cell line chronically exposed to nicotine versus vehicle control. Additional data is also provided from dynamic trajectory analysis, identifying the most dynamic genes due to chronic nicotine treatment. To produce this dataset, we first performed single cell RNA sequencing from HCC38 cells chronically treated with vehicle or nicotine, followed by scanpy analysis to yield 6 discrete cell clusters at conservative resolution. We then evaluated differential gene expression between chronic nicotine and control cells for each individual cluster or in the whole sample using PyDESeq2. For dynamic trajectory analysis, Velocyto (0.6) was used to estimate the spliced and unspliced counts for each gene between chronic nicotine-treated cells and vehicle, allowing computation of gene velocities. These data are useful for analysing the expression of individual genes or gene velocities either in the whole sample or in the different clusters identified. Since the HCC38 cell line used in these experiments is heterogeneous, including cells with features of stem-like, luminal progenitor-like and more differentiated cells, this dataset allows examination of the conserved as well as disparate gene expression effects of nicotine in different breast cancer cell types. Our dataset has a great potential for re-use given the recent surge in interest surrounding the role tobacco-use plays in breast cancer progression.

Specifications Table

**Table:** 

Subject	Cancer Research
Specific subject area	Our work focuses on the role of tobacco products in breast cancer progression and metastasis.
Data format	Raw
Type of data	Excel spreadsheet
Data collection	Data were collected after comparing expression of each gene in the chronic nicotine treated cells to the levels found in the corresponding cell cluster or whole sample from the vehicle-treated cells with PyDESeq2. The provided Log2 fold changes for each gene (DEG) are listed in the Excel spreadsheet deposited.
Data source location	*Institution:* University of California, San Diego *City/Town/Region:* La Jolla, CA *Country:* USA *Latitude and longitude:* 32.876328, -117.236067
Data accessibility	Repository name: UC San Diego Library Digital Collections Raw data in NCBI: https://www.ncbi.nlm.nih.gov/sra/SRX25630993 https://www.ncbi.nlm.nih.gov/sra/SRX25630994 Data identification number: 10.6075/J04×585F Direct URL to data: https://library.ucsd.edu/dc/object/bb71464553
Related research article	Mugisha, S., Baba, S.A., Labhsetwar, S. et al. S100A8/A9 innate immune signaling as a distinct mechanism driving progression of smoking-related breast cancers. Oncogene (2025). 10.1038/s41388-025-03276-5

## 1 Value of the Data

• The data we provided here added value to our original research article by providing an unbiased view of the genes upregulated by nicotine in HCC38 breast cancer cells. This allowed us to assess potential associations between nicotine’s effects and particular cell types. Using these findings, we were able to narrow our focus to cytokine expression as a particular class of genes impacted by nicotine and assess their effects on tumor progression.
• The Log2 fold changes (Log2FC) provided in this dataset are useful for comparing the relative effects of nicotine on different breast cancer cell populations.
• This dataset may be useful to anyone associated with breast cancer research. The mechanisms underpinning smoking’s effects on breast cancer progression are little understood. Additionally, other sources of nicotine, such as e-cigarettes are growing in use and popularity, particularly among young adults. Thus, there is a great need to further elucidate the mechanisms associated with nicotine’s effects on tumor progression, also this dataset may aid future studies of this topic.
• These data can also be used for further insights into associations between chronic nicotine exposure and numerous additional variables, including cell-of-origin, mutational status, copy number alterations (CNA), subtype, etc.

## 2 Background

Tobacco-use is associated with an increased risk of breast cancer progression. In addition to enhancing the risk of developing breast cancer, strong clinical evidence also links smoking with a significantly worse outcome in patients already diagnosed with the disease [1,2]. Despite the discovered clinical link between tobacco-use and breast cancer progression, the cause is still relatively unknown. This is mainly due to a lack of understanding of the mechanisms responsible for smoking’s effects. In fact, the level at which nicotine directly alters breast cancer cell behavior remains largely unexplored. Previous studies of nicotine’s effect in breast cancer cells have yielded only modest and transient alterations on EMT markers and cell proliferation [[3], [4], [5], [6]], leading some to speculate that its primary impact may be on the tumor microenvironment [[7], [8], [9]]. In our published original article, we used unbiased approach to generated a significant difference in innate immune gene expression reprogramming due to chronic nicotine, providing a novel molecular basis of smoking-related breast cancers.

## 3 Data Description

The deposited dataset consists of one Excel spreadsheet listing 8 different worksheet tabs labelled and described as follow:

DEG – The top of each column of the sheet-tabs #2–7 indicates Log2FC and associated adjusted P-values of corresponding downregulated genes in vehicle compared to chronic nicotine-treated cells in each cluster or the overall sample. Presented Log2FC scores for respective genes were obtained via differential gene expression by using PyDESeq2, and data are ranked by Log2FC with a cut-off of 0.5. Additional columns include baseMean, representing the average of the normalized count values across all samples, log2 fold change standard error (lfcSE), Wald statistic (stat) representing Log2FC divided by its standard error, *p*-value and adjusted p-value (padj) generated using the Wald statistical test. The resulting data were processed into Excel format.

Trajectory analysis – The worksheet tab #8 identifies the list of the most dynamic genes ranked from high to low with their corresponding clusters indicated on the top of each column in chronic nicotine-treated cells versus control-vehicle. For dynamic trajectory analysis, Velocyto was used to generate gene-specific velocities and associated genes, before drafting the resulting data into Excel spreadsheet.

Genes are ranked in order of velocities from high to low.

## 4 Experimental Design, Materials and Methods

HCC38 cells used in this study were purchased from ATCC (Manassas, VA, USA). The cells were cultured in complete DMEM medium and maintained in a humid incubator at 37 ⁰C supplied with 5 % CO2. We seeded 3 × 10^6^ cells into 10 cm petri-dishes before adding 100 nM of nicotine (N3876-5ML, Sigma-Aldrich, Saint Louis, MO, USA) every other day for 14 days, with passages performed every 3 days. In a second dish of cells, we concurrently administered an equal volume of vehicle (ethanol) to generate appropriate controls. Cells were passaged 3 times post nicotine treatment prior to library preparation. To prepare sc-RNA-seq libraries, we used 1 × 10^4^ HCC38 cells chronically treated with vehicle or nicotine following the 10X Genomics Single Cell 3′ V2 Reagent Kits User Guide PN-120233 (10X Genomics, Pleasanton, CA, USA). Libraries were then submitted to the UCSD Institute for Genomic Medicine (IGM) Genomics Core for validation of quality and sequencing on a NovaSeq 6000 (Illumina, San Diego, CA, USA).

To process the sc-RNA-seq data, we used the Cell Ranger toolkit (version 3.1.0) provided by 10x Genomics. We performed dimension reduction and unsupervised clustering according to the standard workflow in Scanpy [10]. Principal component analysis (PCA) was then performed on top 8000 highly variable genes (HVGs) to reduce noise, followed by cell clustering using Leiden algorithm [11]. For differential gene expression analysis, we applied PyDESeq2, a re-implementation of DESeq2 in Python [12]. PyDESeq2 estimates variance-mean dependence in count data from sequencing assays and tests for differential expression based on a model using the negative binomial distribution. *P* < 0.05 was considered statistically significant. Additionally, we performed dynamic trajectory analysis using Velocyto (0.6) to estimate the spliced and unspliced counts from the pre-aligned bam files. The dynamical velocity model from scVelo (0.2.2) was applied to calculate RNA velocity, latent time, root, and terminal states. The output was then processed into Excel format and saved as final results files.

## Limitations

None.

## Ethics Statement

We confirm that the authors have read and follow the ethical requirements for publication in Data in Brief and that the current work does not involve human subjects, animal experiments, or any data collected from social media platforms.

## CRediT authorship contribution statement

**Samson Mugisha:** Conceptualization, Methodology, Formal analysis, Writing – original draft. **Shreyas Labhsetwar:** Methodology, Formal analysis, Software. **Devam Dave:** Methodology, Formal analysis, Software. **Richard Klemke:** Methodology, Formal analysis, Software. **Jay S. Desgrosellier:** Conceptualization, Methodology, Formal analysis, Visualization, Investigation, Supervision, Funding acquisition.

## Data Availability

University of California San Diego Library Digital CollectionsA dataset of chronic nicotine-induced genes in HCC38 breast cancer cells (Original data)
